# Editorial

**DOI:** 10.1093/rb/rbu004

**Published:** 2014-10-20

**Authors:** Xingdong Zhang, Fu-Zhai Cui

**Affiliations:** Editor-in-Chief; Executive Editor-in-Chief

In recent years, biomaterials have advanced into a burgeoning interdisciplinary research field combining traditional material science, engineering, biology and medicine. Over the last half-century, many types of lifeless materials such as metals, ceramics and polymers have become ‘bio’-materials, making their way into the fabrication of medical devices for the repair or replacement of damaged or deteriorating human tissues and organs. As we proceed into this new century, this process has accelerated. In the last decade alone, the integration of advances in materials and life sciences with cutting-edge medical sciences and nanotechnologies has driven biomaterial research into an explosive phase. In our pursuit of happiness, longevity and quality of life, we aspire to acquire abilities not only to repair damaged tissues and organs, but also to regenerate them. Driven by this desire and the advances in stem cell and developmental biology, biomaterials science has entered a ‘regenerative’ era. A new crop of materials has gained regenerative properties in becoming ‘smart biomaterials’ that can not only provide mechanical support, but also function as sophisticated regulators of biological activities by activating stem cells and triggering the body’s own regenerative processes to restore functions of damaged tissues and organs.

The potential of regenerative biomaterials is limitless. Even traditionally considered non-regenerative tissues such as nerves and teeth can now regenerate with the help of advanced biomaterials and stem cells. Materials with regenerative properties have laid the foundation upon which regenerative medicine and stem cell therapies can be built, and spawned a new research field of ‘regenerative biomaterials’, which encompasses a variety of research areas including stem cell therapy, tissue engineering and biomedical engineering. Many scientists from these diverse disciplines have been attracted to this rapidly expanding field, bringing in new ideas, new approaches and a new flood of research articles on this red-hot topic. Against this backdrop, we are launching ‘*Regenerative Biomaterials*’, a Chinese Society for Biomaterials (CSBM) journal and a platform devoted to disseminating the latest research advances in regenerative biomaterials as well as traditional biomaterials and medical devices.

Founded in April 2012, the CSBM is a non-profit academic organization for Chinese biomaterial researchers inside China and abroad. The CSBM is one of the six founding members of the International Union of Societies for Biomaterials Science and Engineering (IUSBSE). In June 2012, CSBM successfully organized the 9th World Biomaterials Congress in Chengdu, which received great attendance and applause from scientists worldwide. As we all know, almost all influential societies in the field of biomaterials have their official journals, such as the *Journal of Biomedical Materials Research* (Part A and Part B, published by Wiley), the official journal of the Society for Biomaterials (USA), the Japanese Society for Biomaterials, the Australasian Society for Biomaterials and the Korean Society for Biomaterials; the *Journal of Materials Science: Materials in Medicine* (published by Springer), the official journal of the European Society for Biomaterials; and *Biomaterials Research* (published by BioMed Central), the official journal of the Korean Society for Biomaterials. Therefore, it is quite necessary for the CSBM to have its own official journal for nurturing the development of Chinese biomaterial research and cultivating interaction with scientists from other parts of the world. In addition, the ever-growing number of high-quality manuscripts submitted annually from China also propels the need of a new journal and guarantees the standard of our publication. To forge international collaboration, we also accept high-quality manuscripts from scientists worldwide.


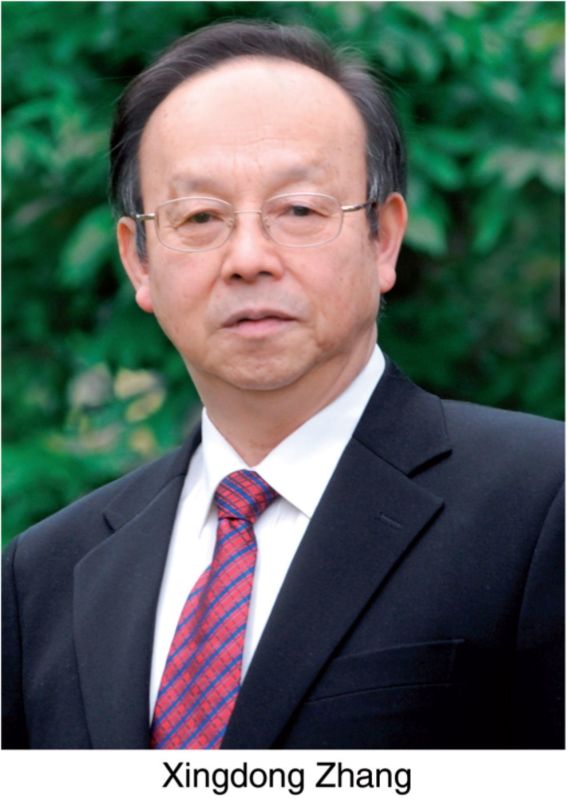



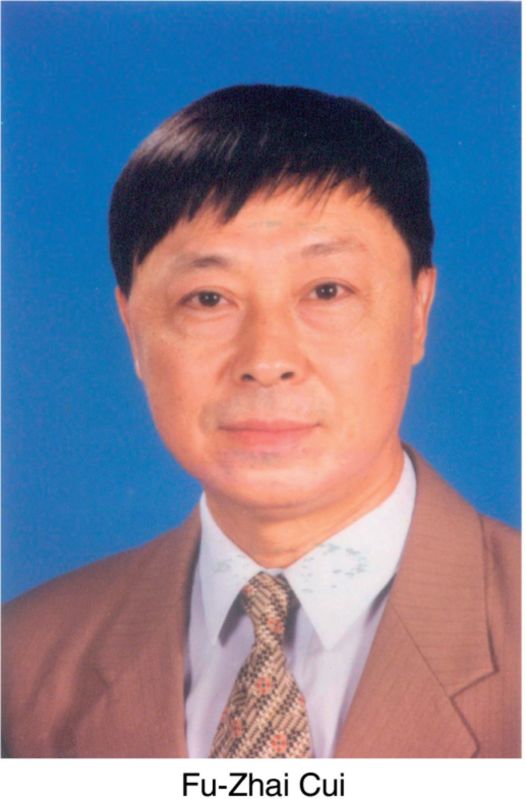


*Regenerative Biomaterials* will be an international, interdisciplinary, peer-reviewed journal concerning frontiers and advances in the new generation of biomaterials and regenerative medicine. The aim of the journal is to provide a forum for original research papers, reviews, clinical case reports and commentaries on the topics relevant to the development of advanced regenerative biomaterials. Manuscripts using novel regenerative technologies and therapeutic approaches for the regeneration and repair of damaged tissues and organs, and manuscripts addressing interactions between biomaterials and cells or tissue, especially stem cells, will be considered with high priority. However, studies on cells or stem cells in regenerative medicine but without materials will not be included. The journal will be a platform for communication among various disciplines concerning biomaterials; these include nanotechnologies, stem cell biology, regenerative medicine, and clinical medicine. We hope that the new journal will contribute to the world community of biomaterials with rapid publication of high-level, creative research.

